# Chasing rabies herd immunity: evaluating dog vaccination strategies and post-vaccination survey reliability in urban and peri-urban Bangladesh

**DOI:** 10.3389/fmicb.2025.1696281

**Published:** 2026-01-20

**Authors:** Catherine Swedberg, Umme Ruman Siddiqi, Ravikiran Keshavamurthy, Md Sohel Rana, Kamrul Islam, Erin Kennedy, Yasmeen B. Ross, Sarah Bonaparte, Frederic Lohr, Hasan Sayedul Mursalin, Md Kamruzzaman, Luke Gamble, Andrew D. Gibson, Jesse D. Blanton, Ryan M. Wallace

**Affiliations:** 1 US Centers for Disease Control and Prevention, Atlanta, GA, United States; 2 Worldwide Veterinary Service, Cranborne, United Kingdom; 3 Directorate General of Health Services, Ministry of Health and Family Welfare, Dhaka, Bangladesh; 4 Department of Livestock Services, Ministry of Fisheries and Livestock, Dhaka, Bangladesh

**Keywords:** dog-mediated rabies, dog vaccination programs, human to dog ratios, population enumeration, post-vaccination evaluation, rabies control, dog density

## Abstract

**Introduction:**

Mass dog vaccination (MDV) is essential for eliminating dog-mediated rabies, responsible for over 95% of the estimated 74,000 annual human rabies deaths. Achieving ≥ 70% vaccination coverage necessary for herd immunity remains challenging, underscoring the need for effective vaccination strategies and reliable post-vaccination evaluation methods.

**Methods:**

MDV campaigns were conducted in four site in Bangladesh (two urban, two peri-urban) using three strategies: standard capture-vaccinate-release (CVR), enhanced CVR (eCVR), and roaming static point (RSP). Post-vaccination sight re-sight (SRS) and household surveys were used to characterize dog populations and estimate coverage. Three enumeration methods were compared to assess reliability and utility for campaign evaluation.

**Results:**

Over 12 working days, 9,195 dogs were vaccinated. eCVR achieved the highest operational efficiency (40.2 dogs/vaccinator/day), followed by standard CVR (36.6) and RSP (18.2). Post-vaccination surveys indicated that over 93% of dogs were free-roaming, and around 30% were unowned, highlighting limitations of static point strategies. Human-to-dog ratios (HDRs) were lower than the 100:1 planning estimate and varied widely across sites (mean: 67.8; range: 21.1–129.6), with no significant difference between urban and peri-urban areas (*p* = 0.479). Coverage estimates differed by enumeration method: 19% (dog density), 32% (HDR), and 47% (SRS), with comparable SRS- and HDR-based estimates (*p* = 0.920), and dog density formula estimates significantly lower (*p* = 0.014). Averaging across enumeration methods and sites, eCVR achieved the highest coverage (45%), followed by standard CVR (37%) and RSP (16%), with estimated RSP coverage significantly lower than eCVR (*p* = 0.028).

**Discussion:**

The wide heterogeneity in HDRs highlights the limitations of using a single ratio for national dog population extrapolation. Effective rabies control requires locally tailored vaccination strategies, real-time monitoring, and robust enumeration techniques to guide planning and ensure reliable evaluation of campaign impact.

## 1 Introduction

Since the 1920s, mass dog vaccination (MDV) has been a cornerstone global strategy to eliminate the dog-maintained rabies virus variant, by interrupting transmission and reducing the number of rabid dogs and associated human exposures (Lankester et al., 2014; Umeno, 1921). To establish herd immunity and prevent outbreaks, achieving and maintaining at least 70% vaccination coverage among free-roaming dogs is essential, with spatial uniformity necessary to avoid pockets of susceptibility where the virus can persist (Coleman and Dye, 1996; Ferguson et al., 2015). Despite these well-established principles, dog-mediated rabies remains responsible for over 95% of the 74,000 annual human rabies deaths estimated worldwide (World Health Organization [WHO], 2018).

Bangladesh bears one of the highest global rabies burdens, with an estimated 1,010–2,200 human deaths each year (Bonaparte et al., 2023). In response, the government launched the National Rabies Elimination Programme in 2011, adopting a multifaceted strategy that includes MDV and expanded access to post-exposure prophylaxis (PEP) (Acharya et al., 2021). Over the past decade, PEP availability has increased widely, with vaccines and immunoglobulin provided free of charge at central, district, and sub-district health facilities (Sreenivasan et al., 2019). Despite these advances, rabies remains endemic, driven by inconsistent dog vaccination coverage (Ghosh et al., 2024) and a high annual bite incidence of 628 per 100,000 persons, with nearly half of bites classified as moderate or high risk, and incomplete wound washing and PEP initiation among high-risk bite victims (Ross et al., 2022). National expenditure on PEP is estimated to exceed USD 4.5 million annually to support approximately 250,000 PEP regimens (Li et al., 2019; Ross et al., 2022). Collectively, these findings highlight the critical need for high-coverage MDV to prevent human rabies deaths, even in settings where PEP is broadly accessible (Lavan et al., 2017; Swedberg et al., 2023).

Achieving adequate coverage (>70%) in low- and middle-income countries (LMICs) remains challenging, particularly during initial MDV campaigns (Taylor et al., 2017). Strategies must be tailored to local dog population demographics to maximize coverage among susceptible dogs. Static point (SP) and door-to-door (DD) methods are effective where dogs are primarily owned (Gibson et al., 2016) but are less suitable in areas with predominantly unowned or loosely owned free-roaming dogs. In such contexts, capture-vaccinate-release (CVR) by trained teams is often required to reach hard-to-catch dogs. At the time of this study, CVR was the standard strategy in Bangladesh and was implemented without real-time data technologies. From 2018 to 2023, the national MDV program reported mean district-level coverage exceeding 80% where campaigns were conducted; however, in any given year, at most about half of the country’s 64 districts were vaccinated. In 2019, the program vaccinated the highest number of dogs across 33 districts (*n* = 625,208, 37.5% of the estimated national population of 1,668,140), whereas only 13 districts conducted MDV in 2023 (Ghosh et al., 2024).

In most LMICs, dog vaccination through the private veterinary sector is uncommon, placing the onus on government-led programs to reach susceptible dogs for rabies control (Cleaveland et al., 2018). Yet, effective operational planning and campaign evaluation are often hindered by a lack of reliable baseline data on dog population size, distribution, and characteristics (Taylor et al., 2017; Wallace et al., 2019). While national dog censuses are sometimes conducted for dog enumeration, these are costly, time-intensive, and fail to account for unowned or community dogs, which are at high risk of rabies. More frequently, planners of rabies control programs rely on a single human-to-dog ratio (HDR) derived from a small, localized study to extrapolate national dog populations. However, given the variability in dog ownership practices across regions, influenced by factors such as urbanization and religion, this approach often yields inaccurate and non-representative estimates, typically underestimating the dog population (Chazya et al., 2025; Moran et al., 2022).

Studies in Bangladesh report highly variable HDRs, ranging from 828: 1 in urban Dhaka (52 dog/km^2^) (Tenzin et al., 2015) to 120: 1 in rural areas (14 dogs/km^2^) (Hossain et al., 2013), to a lower national average HDR of 86.7: 1 (12.8 dogs/km^2^) (Ghosh et al., 2024). These discrepancies reflect both contextual differences and the lack of standardized enumeration methods, complicating the reliability of post-vaccination coverage estimates. A well-established approach for estimating dog populations combines sight re-sight (SRS) and household surveys (HHS), which, when conducted alongside campaigns, enable more accurate assessments of dog population size for evaluating coverage and operational strategies (Chazya et al., 2025; Moran et al., 2022). However, SRS and HHS require strict adherence to sampling protocols, including systematic transects that extend beyond main roads into peripheral community pathways to ensure a representative survey population (Meunier et al., 2019).

This study aimed to inform the design of effective MDV campaigns and to evaluate the reliability of post-vaccination survey data. Campaigns were conducted in two urban and two peri-urban sites in Bangladesh, using three vaccination strategies: standard CVR, enhanced CVR, and roaming static point. Performance was assessed through vaccination coverage and operational efficiency (daily vaccination rate), enabling comparisons across sites and strategies. Post-vaccination coverage was estimated using three dog population enumeration methods (HDR-based, a dog density formula, and SRS-based estimates), and each approach was subsequently evaluated for its reliability and utility for campaign evaluation.

## 2 Materials and methods

### 2.1 Study locations

**FIGURE 1:**
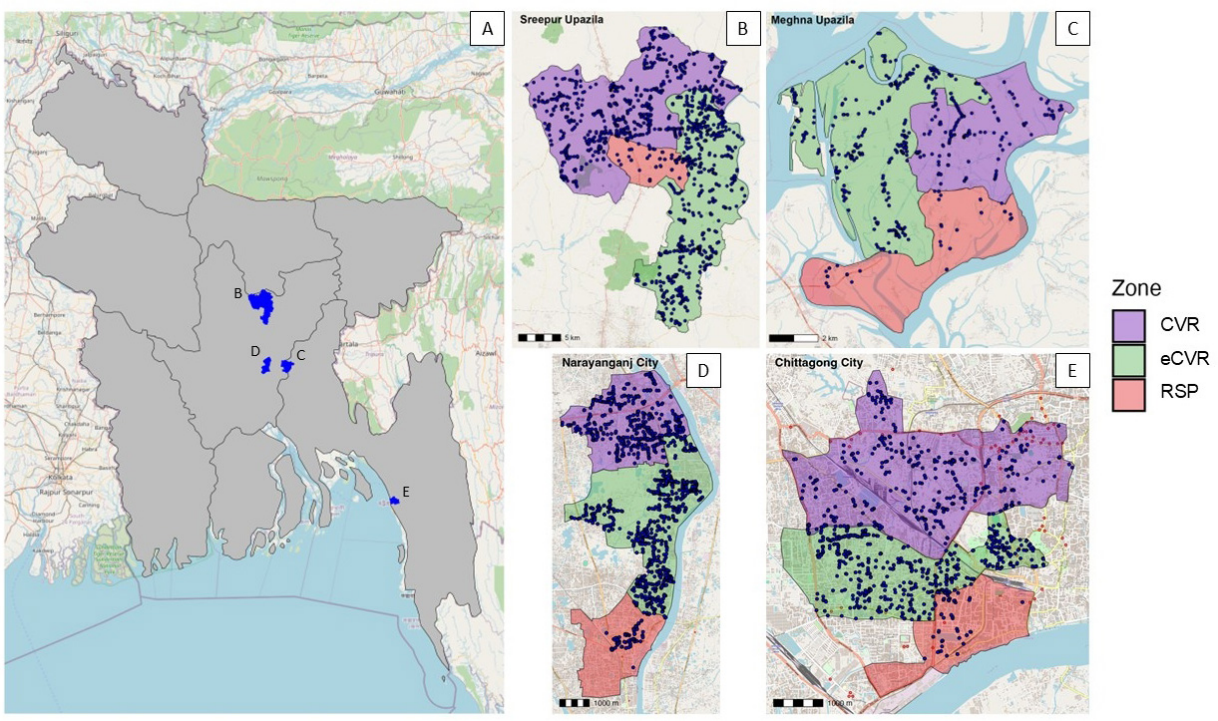
Map of Bangladesh showing vaccination locations and distribution of dog vaccination points (black dots) by site. **(A)** National map with districts selected for vaccination highlighted in blue. **(B–E)** Study sites with dog vaccination points: **(B)** Sreepur Upazila (peri-urban) study site in Dhaka Division. **(C)** Meghna Upazila (peri-urban) study site in Chittagong Division. **(D)** Narayanganj City (urban) study site in Dhaka Division. **(E)** Chittagong City (urban) study site in Chittagong Division. Polygon boundaries were sourced from UN-OCHA Humanitarian Data Exchange (United Nations Office for the Coordination of Humanitarian Affairs [OCHA], 2025), and base layer maps from OpenStreetMap (OpenStreetMap [OSM], 2025).

Rabies MDV campaigns were conducted and evaluated across four sites in Bangladesh from July to September 2018. Two of the country’s eight administrative divisions, Dhaka and Chittagong, were selected for inclusion. Within each division, one urban site (population density > 40,000 persons/km^2^) and one peri-urban site (population density ∼1,000 persons/km^2^) were chosen. In Dhaka Division, the selected sites were Narayanganj City (urban) and Sreepur Upazila (peri-urban), while in Chittagong Division, Chittagong City (urban) and Meghna Upazila (peri-urban) were selected (Figure 1).

### 2.2 Study design

Vaccination campaign protocols were developed by the International Rabies Taskforce (IRT), including the US Centers for Disease Control and Prevention (US-CDC) and Worldwide Veterinary Service (WVS), in collaboration with the Bangladesh Directorate General of Health Services (DGHS) and the Department of Livestock Services (DLS). Campaign sites were selected based on national census data and outcomes of previous campaigns in alignment with Bangladesh’s strategic rabies control priorities.

Three vaccination methods were evaluated: (1) standard CVR, the routine method used in Bangladesh at the time; (2) enhanced CVR (eCVR), which incorporated real-time data monitoring to guide daily operations; and (3) roaming static point (RSP), where temporary clinics relocated every 2–3 hours (or once demand declined) to predetermined sites selected by DGHS in easily accessible public community spaces (e.g., markets, schools, mosques).

The four study sites described above (two urban, two peri-urban) were each divided into three zones, with each zone containing an estimated 200 free-roaming dogs based on the official HDR of 100: 1 used in Bangladesh at the time. Each zone was randomly assigned one of the three vaccination methods, resulting in a total of 12 vaccination zones in the study (Figures 1B–E).

Campaigns at each site included two to three days of planning, training, and community mobilization, followed by five to six days of vaccination, and two days of post-vaccination evaluation. In eCVR zones, teams were supported by real-time data and operational guidance from international partners.

Vaccination teams included DLS and DGHS staff, with composition varying by vaccination method. Both standard CVR and eCVR teams comprised five members (one vaccinator, two dog catchers, one porter, and one data collector), whereas RSP teams were smaller, comprising four members (one vaccinator, one dog catcher, one porter, and one data collector). All sites, except Sreepur, deployed four teams for each CVR and eCVR strategies, and two teams for the RSP strategy. Sreepur, which covered a larger geographic area, deployed eight CVR and eight eCVR teams, along with two RSP teams. All vaccinated dogs were marked with color-coded wax paint to identify the vaccination method used.

Post-vaccination evaluations were conducted by independent survey teams to reduce bias. Household surveys (HHS) were used to estimate vaccination coverage and characterize owned dog populations, while sight re-sight (SRS) surveys were used to assess these parameters among free-roaming dogs.

### 2.3 Data collection

#### 2.3.1 Vaccination campaign

All vaccination teams used the WVS smartphone application to gather data during campaigns. Recorded variables included the time, date, GPS coordinates, project site, and zone (CVR, eCVR, RSP) for each vaccinated dog. Transect data were also captured to document the route walked by each team.

#### 2.3.2 Post-vaccination evaluation

**SRS protocols:** Survey teams, each consisting of two persons (one dog counter and one data recorder), conducted SRS surveys across all 12 vaccination zones to: (1) estimate the free-roaming dog population size, and (2) assess post-vaccination coverage among free-roaming dogs, following previously published methodologies (Chazya et al., 2025; Moran et al., 2022). Dogs were categorized according to the Lincoln-Peterson formula, with data collected for Day 1 sightings (*n1*), Day 2 sightings (*n2*), and Day 2 repeat sightings (*n2R*) (Amaral et al., 2014; Léchenne et al., 2016).

Surveys were conducted over two consecutive days using the WVS App to record dog sightings and GPS-tracked transect pathways. The same procedure was followed on both days to document newly sighted dogs and those previously recorded. For each dog observed, data were recorded, including the GPS location, presence of a vaccination mark, and distinctive physical characteristics for dog identification.

To minimize bias, survey teams were not informed of vaccination activity locations to prevent intentional overlap that could lead to overestimated coverage within a zone. Surveyors were instructed to systematically walk each zone using the adjacent strip transect method to ensure comprehensive spatial coverage, guided by polygons of the evaluation zones displayed on the WVS App.

**HHS protocols:** Cross-sectional HHS were conducted across all 12 evaluation zones using a multi-stage cluster sampling design. Teams of two interviewers were instructed to follow SRS transects within each zone, interviewing every 40th household in urban areas and every 16th household in peri-urban areas. A standardized questionnaire was administered to consenting adults (≥ 18 years) to record household demographics, number of owned dogs, and each dog’s vaccination and roaming status. Dogs were categorized by roaming behavior as always roaming (*ARD*_*HHS*_), sometimes roaming (*SRD*_*HHS*_), or confined (*CD*_*HHS*_).

Surveys were designed to serve a dual purpose of evaluating the vaccination campaign and collecting knowledge, attitude, and practices (KAP) data on rabies healthcare-seeking behaviors (Ross et al., 2022). Sample size was determined using the cohort methodology described in Fleiss et al. (2013), assuming a 5% anticipated bite rate and applying a continuity correction. This resulted in an estimated 660 household surveys per site, totalling 2,640 across the four study sites.

### 2.4 Data analysis

#### 2.4.1 Dog population size and demographics

To estimate the overall dog population size and demographics (i.e., ownership and roaming status), SRS and HHS data were combined using a stepwise approach previously described in other rabies MDV studies (Chazya et al., 2025; Moran et al., 2022).

**SRS database:** Human population and geographic area (km^2^) data were obtained from the Global Human Settlement Layer (GHSL) project (Global Human Settlement Layer [GHSL], 2020). Geospatial data were extracted using the Simple Features (*sf*) and *terra* packages in R Studio (Version 4.3.1). Zone polygon shapefiles were used to calculate human population density for each site. A sightline buffer of 100 meters was added to SRS transects, and the transect length (km), area (km^2^), and human population along the transect were calculated.

To estimate the number of free-roaming dogs (*FRD*_*SRS*_) observed during SRS surveys, geospatial data were analyzed with SRS counts (*n1* = *Day1 sighting*, *n2* = *Day2 sighting*, *n2R* = *Day2 re-sighting*) applying the Lincoln-Peterson formula with Chapman’s bias correction (Chapman, 1951):


$$F\,R\,D_{S\,R\,S}=\left[\frac{\left(n\,1+1\right)\,\left(n\,2+1\right)}{n\,2\,R+1}-1\right]$$

Seber’s formula (Seber, 1970) was then applied to calculate unbiased variance of the free-roaming dogs estimate (*FRD*_*SRS*_), and used to derive 95% confidence intervals (CIs):


$$V\,a\,r\,\left(F\,R\,D_{S\,R\,S}\right)=\left[\frac{\left(n\,1+1\right)\,\left(n\,2+1\right)\,\left(n\,1-n\,2\,R\right)\,\left(n\,2-n\,2\,R\right)}{{\left(n\,2\,R+1\right)}^{2}\,\left(n\,2\,R+2\right)}\right]$$

$$95\%\,C\,I=F\,R\,D_{S\,R\,S}\pm 1.965\,\sqrt{V\,a\,r\,\left(F\,R\,D_{S\,R\,S}\right)}$$

To estimate the total number of free-roaming dogs in each community (*FRD*_*Total*_), SRS counts were adjusted based on the sampling fraction of the human population represented by the transect area and extrapolated to the total population of each site. Free-roaming HDRs were then calculated using *FRD_*Total*_* and the total human population.

**HHS database:** Owned dog population size was estimated for both confined dogs (*OCD*_*HHS*_) and free-roaming dogs (*OFRD*_*HHS*_ = *ARD*_*HHS*_+*SRD*_*HHS*_) at each site by dividing the dog count by the household study population, extrapolated to the total community (site) population. The total number of owned dogs (*OD*_*Total*_) was calculated as:


$$O\,D_{T\,o\,t\,a\,l}=O\,C\,D_{T\,o\,t\,a\,l}+O\,F\,R\,D_{T\,o\,t\,a\,l}$$

95% CIs were calculated for all estimates. HDRs were derived using total owned dog estimates and the total household population per site.

**Population analysis:** SRS and HHS data were merged into a single dataset, with validation conducted to identify inconsistencies. HHS estimates were considered valid if *ARD*_*HHS*_ was less than the upper bound of *FRD*_*SRS*_ 95% CI. SRS estimates were considered valid if *FRD*_*SRS*_ exceeded the lower bound of *FRD*_*HHS*_ 95% CI.

Overall dog population demographics were calculated following methods described in Moran et al. (2022). SRS estimates of total free-roaming dogs (*FRD*_*Total*_) and HHS estimates of owned, free-roaming dogs (*OFRD*_*Total*_) were combined to calculate the total number of unowned community dogs (*UCD*_*Total*_), which was used to calculate the total dog population:


$$T\,o\,t\,a\,l,U\,n\,o\,w\,n\,e\,d\,C\,o\,m\,m\,u\,n\,i\,t\,y\,D\,o\,g\,s\,\left(U\,C\,D_{T\,o\,t\,a\,l}\right)=$$

$$\max\,\left\{\left(F\,R\,D_{T\,o\,t\,a\,l}-O\,F\,R\,D_{T\,o\,t\,a\,l}\right),0\right\}$$

$$T\,o\,t\,a\,l\,D\,o\,g\,P\,o\,p\,u\,l\,a\,t\,i\,o\,n=O\,D_{T\,o\,t\,a\,l}+U\,C\,D_{T\,o\,t\,a\,l}$$

Regression model analyses were performed in R to assess associations between human population density {derived from GHSL raster data (Global Human Settlement Layer [GHSL], 2020)} and survey-derived indicators: total HDR, free-roaming HDR, total dog density, and free-roaming dog density. Each of these associations was tested using linear, logarithmic, exponential, and power models, with model fit evaluated by the highest R^2^ value.

#### 2.4.2 Vaccination coverage

Coverage for each vaccination method (CVR, eCVR, RSP) and site was calculated as the proportion of dogs vaccinated, as recorded in the WVS App, relative to the estimated dog population. Coverage estimates were derived using three enumeration approaches: HDR-based, dog density formula, and SRS surveys.

For each vaccination method and enumeration approach, mean coverage, standard deviations (SD), and 95% CIs were calculated. Pairwise comparisons were performed using two-sample *t*-tests, with SRS serving as the reference enumeration method and eCVR as the reference vaccination strategy. For each comparison, the *p*-value and 95% CI for the mean difference were reported to assess statistical significance.

Vaccinator efficiency was calculated as the mean daily vaccination rate (dogs vaccinated per vaccinator per day) and summarized by method, site, and urbanicity, with corresponding 95% CIs. Days with fewer than five dog vaccinations were excluded to avoid skewing estimates.

#### 2.4.3 Post-vaccination data validation

Vaccination routes were reconstructed using GPS coordinates of vaccination points and the Open Source Routing Machine (*osrm*) package in R. Consistent with the method used for SRS transects, vaccination routes were buffered (100 m) to account for vaccinator movement covering houses and dogs within their line of sight, then total route length (km) and total area covered (km^2^) were calculated.

Effective post-vaccination surveys should achieve broad and representative spatial coverage of the campaign evaluation zone while avoiding overrepresentation of vaccinated areas. Accordingly, optimal survey design is characterized by high spatial coverage, irrespective of the degree of overlap with vaccination routes. In contrast, low spatial coverage with high overlap is likely to overestimate coverage, while low spatial coverage with low overlap may underestimate coverage.

To evaluate survey data reliability, two metrics were compared in each zone: (1) spatial coverage, defined as the proportion of the evaluation zone traversed by SRS field team transects, and (2) spatial overlap, defined as the area (km^2^) of overlap between SRS transects and vaccination routes.

Based on these comparisons, a reliability rating was calculated for each zone as a ratio of vaccination spatial coverage to spatial overlap. Ratings indicated low (< 0.5), moderate (0.5–1.0), or high (> 1.0) confidence in vaccination coverage estimates. Zones with high reliability ratings were considered to have met ideal survey design criteria (i.e., high spatial coverage, with moderate overlap with vaccination routes), while low reliability ratings suggested potential bias from over- or underestimation of coverage.

## 3 Results

### 3.1 Vaccination campaign

Over 12 working days in 2018, a total of 9,195 dogs were vaccinated across four study sites in Bangladesh: Chittagong (2,312), Meghna (1,152), Narayanganj (2,654), and Sreepur (3,077). Vaccinations were delivered through three strategies, including standard CVR (4,000 dogs; 43.5%), eCVR (4,391 dogs; 47.8%) and RSP (804 dogs; 8.7%).

**FIGURE 2:**
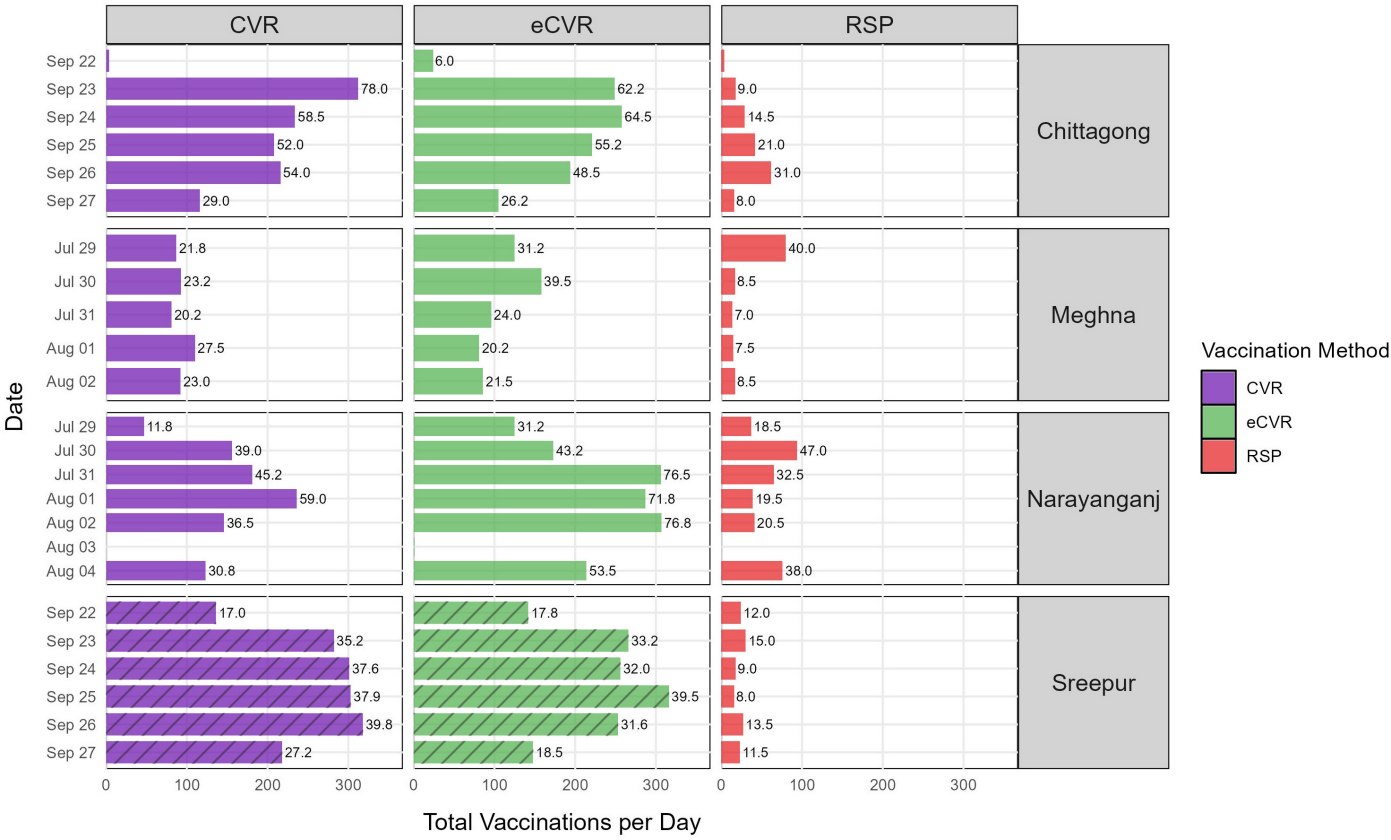
Daily number of dogs vaccinated per zone by study site (rows). Mean efficiency (dogs vaccinated per vaccinator per day) is shown for days with five or more dogs vaccinated. All sites deployed four teams in CVR and eCVR zones, except Sreepur, which deployed eight teams (striped bars). Two teams were deployed in RSP zones at all sites. CVR, standard capture-vaccinate-release; eCVR, enhanced capture-vaccinate-release; RSP, roaming static point.

Vaccination efficiency, defined as the number of dogs vaccinated per vaccinator per day, varied across strategies. The eCVR strategy achieved the highest mean daily efficiency (40.2 dogs/vaccinator/day; SD = 4.2; 95% CI: 32.0–48.4), followed by standard CVR (36.6; SD = 3.4; 95% CI: 29.8–43.3) and RSP (18.2; SD = 2.6; 95% CI: 13.2–23.2) (Figure 2). Urban sites demonstrated higher efficiency than peri-urban sites, with mean rates of 40.3 dogs/vaccinator/day (max = 78; SD = 3.6; 95% CI: 33.2–47.5) compared with 23.0 (max = 40; SD = 1.9; 95% CI: 19.3–26.8), respectively. Among individual sites, Narayanganj recorded the highest mean efficiency (41.7 dogs/vaccinator/day), while Meghna reported the lowest (21.6).

### 3.2 Post-vaccination surveys

Across the four study sites, the total community population was 2,543,527, covering an area of 584.1 km^2^. Field teams conducted post-vaccination surveys (both SRS and HHS) in all 12 evaluation zones. In total, 2,442 household surveys were completed, representing a surveyed population of 13,536 people and 182 owned dogs. For SRS, field teams walked a cumulative 463.4 linear kilometers over 24 survey days, recording 3,072 unique free-roaming dogs across the two-day survey period (*Day1* = 2,056; *Day2* = 1,946; *Day2Resight* = 930). The total human population within the SRS transects was 470,505 (315,354 in urban and 155,151 in peri-urban), spanning a total area of 48.5 km^2^, or 8.3% of the total study area.

#### 3.2.1 Dog ownership characteristics

Across all sites, the majority of dogs observed were free-roaming (93.2%) and thus susceptible to rabies transmission. Of these, 63.5% were owned free-roaming dogs (OFRD), and 29.7% were unowned community (stray) dogs (UCD). Stratified by urbanicity, the proportion of free-roaming dogs was slightly lower in urban areas (92.7%) than in peri-urban areas (94.1%).

Ownership status showed more pronounced variation. The proportion of owned, free-roaming dogs was lower in urban areas (56.0%) than in peri-urban areas (76.8%), whereas unowned, community dogs were more prevalent in urban areas (36.7%) than in peri-urban areas (17.3%). Owned, confined dogs (OCD), representing a lower-risk group for rabies transmission, accounted for 6.8% of all dogs, with minimal difference by urbanicity (urban: 7.2%; peri-urban: 5.9%).

**FIGURE 3:**
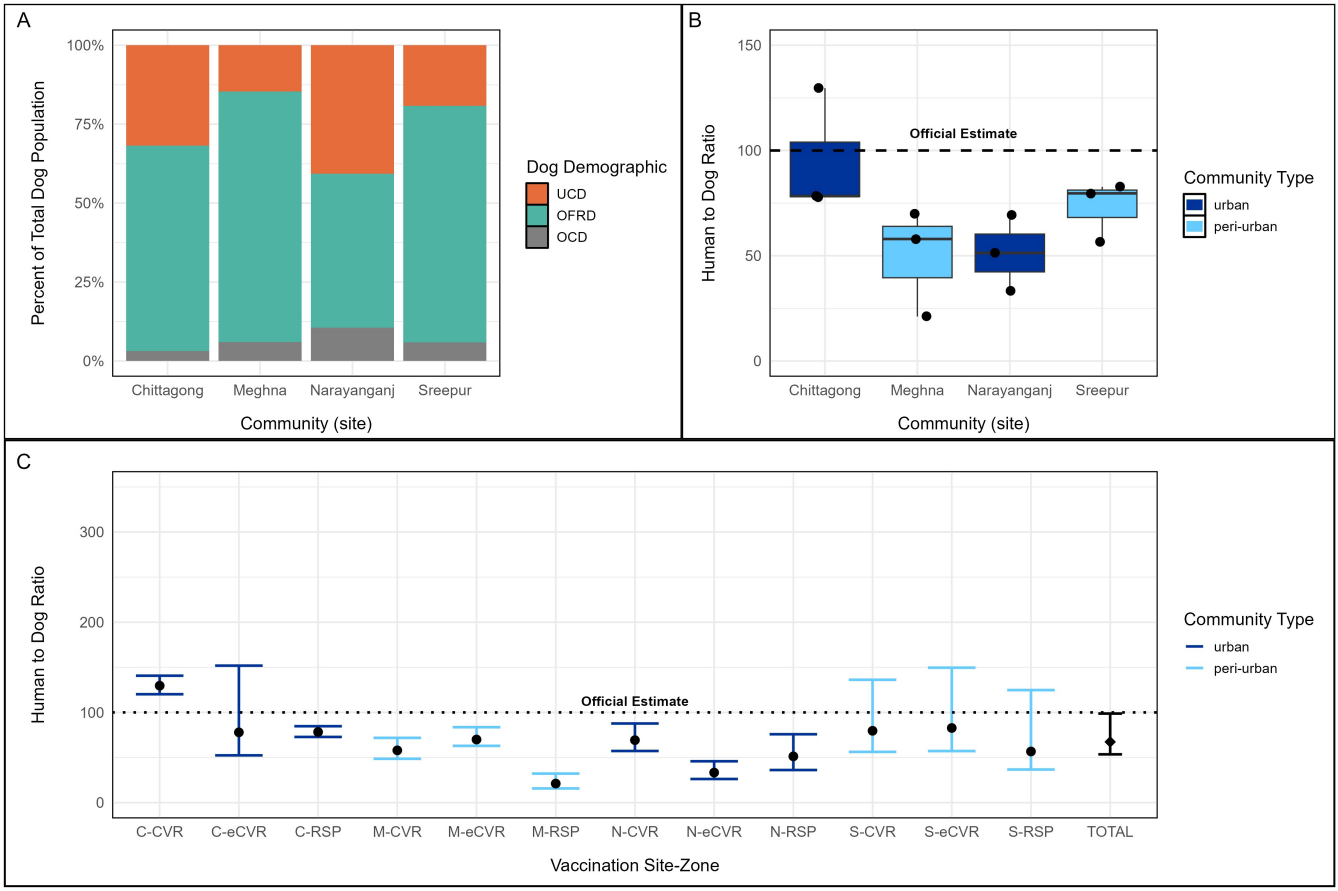
Dog ownership, roaming status, and HDRs across four study sites. **(A)** Dog ownership and roaming status by site. **(B)** Boxplot of HDRs by site and community type (urban = dark blue; peri-urban = light blue). **(C)** HDR comparisons across the 12 vaccination zones. HDR, human: dog ratio.

Variation in ownership practices was also observed across sites within the same urbanicity category (Figure 3A). The proportion of owned free-roaming dogs varied among urban sites (Chittagong: 65.1%, Narayanganj: 48.8%) and peri-urban sites (Meghna: 79.4%, Sreepur: 74.9%). Unowned, community dogs were more prevalent in Narayanganj than in Chittagong (40.7% vs. 31.8%) and in Sreepur than in Meghna (19.2% vs. 14.7%). Owned, confined dogs varied more across urban sites (Chittagong: 3.1%, Narayanganj: 10.5%) than peri-urban sites (both 5.9%).

#### 3.2.2 Dog population estimates

HDRs derived from post-vaccination surveys were consistently lower than the official estimate of 100: 1 used for initial campaign planning, indicating a higher-than-anticipated dog population across study areas (Figures 3B, C). The mean HDR in urban sites was 73.3 (95% CI: 60.8–97.8), with a wide range from 33.3 to 129.6, while peri-urban sites had a slightly lower mean HDR of 61.4 (95% CI: 46.2–99.7), ranging from 21.1 to 82.7 (Table 1). Among individual sites, the highest mean HDR was observed in urban Chittagong (95.3; 95% CI: 81.8–126), followed by peri-urban Sreepur (73.0; 95% CI: 50–137), urban Narayanganj (51.3; 95% CI: 39.9–69.8), and peri-urban Meghna (49.7; 95% CI 42.4–62.5) (Figure 3B). Despite this variation across community types, HDR estimates were statistically similar between urban and peri-urban areas (*p* = 0.479).

**TABLE 1:** Dog population estimates by vaccination method and site, calculated using two different enumeration methods: HDR-based and dog density formula.

Study site	Vaccination method	Area (km^2^)	Human population	Number of dogs vaccinated	Estimated HDR	HDR 95% CI	HDR derived dog population	Estimated dog density	Dog density 95% CI	Density derived dog population
**(A) Summary by site**										
Chittagong	C-CVR	11	508,638	1,090	129.6	120.0–140.7	3,925	418	385–451	4,640
	C-eCVR	7	366,549	1,051	77.9	52.4–151.9	4,705	623	320–927	4,068
	C-RSP	4	57,035	171	78.3	72.8–84.7	728	199	184–214	702
Meghna	M-CVR	21	34,414	463	58.0	48.6–71.8	593	59	48–71	1,233
	M-eCVR	25	53,600	546	70.0	63.0–83.6	766	59	50–66	1,463
	M-RSP	22	29,559	143	21.1	15.7–32.2	1,401	192	126–258	4,186
Narayanganj	N-CVR	9	244,646	889	69.3	57.2–87.7	3,530	470	371–569	4,291
	N-eCVR	14	239,090	1,413	33.3	26.2–45.9	7,180	770	560–980	10,472
	N-RSP	6	195,904	352	51.3	36.1–75.6	3,819	619	419–878	3,894
Sreepur	S-CVR	239	408,155	1,558	79.6	56.3–136.2	5,128	51	30–72	12,189
	S-eCVR	190	192,088	1,381	82.7	57.2–149.6	2,323	41	23–59	7,790
	S-RSP	37	213,849	138	56.7	36.7–124.8	3,772	99	45–154	3,703
All	Total	584	2,543,527	9,195	67.3	53.5–98.7	37,869	300	213–392	58,631
**(B) Summary by vaccination method**										
All	CVR	280	1,195,853	4,000	84.1	70.6–109.1	13,176	250	208–291	22,353
	eCVR	235	851,327	4,391	66.0	49.7–107.7	14,974	373	238–508	23,793
	RSP	69	496,347	804	51.9	40.3–79.4	9,720	277	194–376	12,484

HDR, human: dog ratio.

**FIGURE 4:**
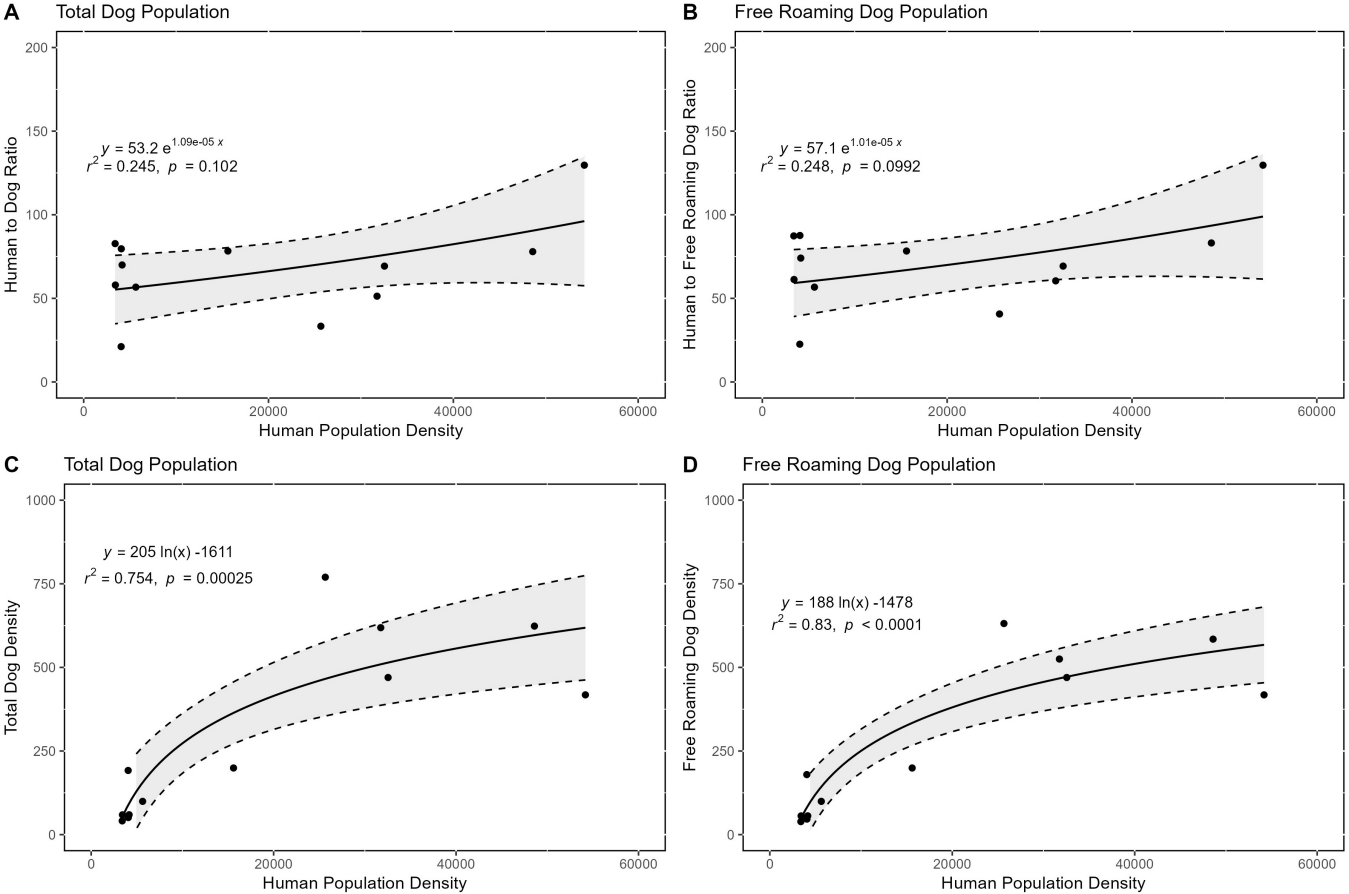
Associations between human population density and dog population metrics: **(A)** total HDR; **(B)** free-roaming HDR; **(C)** total dog density; and **(D)** free-roaming dog density. HDR, human: dog ratio.

Regression models fitted in R showed a weak positive exponential association between human population density and both total HDR (R^2^ = 0.245) and free-roaming HDR (R^2^ = 0.248) (Figures 4A, B). In contrast, strong positive logarithmic correlations were observed between human population density and both total dog density (R^2^ = 0.83) and free-roaming dog density (R^2^ = 0.754) (Figures 4C, D).

#### 3.2.3 Vaccination coverage

Post-vaccination survey data were used to estimate coverage at each site using three enumeration methods: HDR-based estimates, dog density formula, and SRS counts of the proportion of marked (vaccinated) dogs. Coverage varied widely across enumeration methods, vaccination strategies, and sites (Table 2). Across enumeration approaches, the dog-density formula yielded the lowest average coverage (19%; 95% CI: 13.0–25.3), followed by HDR-based estimates (32%; 95% CI: 18.5–45.0), while SRS survey count estimates were highest (47%; 95% CI: 33.8–61.0).

**TABLE 2:** Estimated vaccination coverage calculated from three enumeration methods: HDR-based, dog density formula, and SRS survey data.

Study site	Vaccination method	SRS sighted dogs	SRS vaccinated dogs	SRS coverage	HDR-based coverage	Dog density coverage
**By site and zone**						
Chittagong	C-CVR	549	143	26%	28%	23%
	C-eCVR	333	223	67%	22%	26%
	C-RSP	199	37	19%	23%	24%
Meghna	M-CVR	216	58	27%	78%	38%
	M-eCVR	210	101	48%	71%	37%
	M-RSP	121	15	12%	10%	3%
Narayanganj	N-CVR	394	318	81%	25%	21%
	N-eCVR	348	267	77%	20%	13%
	N-RSP	196	88	45%	9%	9%
Sreepur	S-CVR	212	125	59%	30%	13%
	S-eCVR	208	168	81%	59%	18%
	S-RSP	86	24	28%	4%	4%
All	Total	3,072	1,567	51%	24%	16%
	Average	– – – –	– – – –	47.4%	31.7%	19.1%
	Std Dev			24.0	23.5	10.9
	95% CI			(33.8–61.0)	(18.5–45.0)	(13.0–25.3)
	*p*-value			Ref	0.920	0.014
**By vaccination method**						
All	CVR	1,371	644	47.0%	30.4%	17.9%
	eCVR	1,099	759	69.1%	29.3%	18.5%
	RSP	602	164	27.2%	8.3%	6.4%

Results are presented by site, zone, and average coverage per vaccination method. HDR, human: dog ratio. SRS, sight re-sight survey; CVR, capture-vaccinate-release; eCVR, enhanced capture-vaccinate-release; RSP, roaming static point.

A two-sample *t*-test showed no statistically significant difference between SRS- and HDR-derived coverage estimates (*p* = 0.920), suggesting comparability between these approaches. In contrast, SRS coverage estimates were significantly higher than those derived from dog-density formula (*p* = 0.014), indicating that the density-based method may underestimate coverage, or alternatively, that SRS- and HDR-based methods may overestimate coverage.

These patterns remained consistent when stratified by vaccination strategy. When averaging across enumeration methods and sites, eCVR achieved the highest overall coverage (45%; 95% CI: 31.3–58.6), followed by standard CVR (37%; 95% CI: 25.2–49.6). In contrast, RSP yielded substantially lower coverage (16%; 95% CI: 9.2–22.7), reflecting consistently reduced performance relative to both CVR strategies (Table 3).

**TABLE 3:** Site-specific vaccination coverage by enumeration method and vaccination strategy.

Study site	Enumeration method	Standard CVR (CVR)	Enhanced CVR (eCVR)	Roaming static point (RSP)
Chittagong	C-HDR	28%	22%	23%
	C-DogDens	23%	26%	24%
	C-SRS	26%	67%	19%
Meghna	M-HDR	78%	71%	10%
	M-DogDens	38%	37%	3%
	M-SRS	27%	48%	12%
Narayanganj	N-HDR	25%	20%	9%
	N-DogDens	21%	13%	9%
	N-SRS	81%	77%	45%
Sreepur	S-HDR	30%	59%	4%
	S-DogDens	13%	18%	4%
	S-SRS	59%	81%	28%
All	Average	37.4%	45.0%	15.9%
	Std Dev	21.6	24.2	12.0
	95% CI	(25.2–49.6)	(31.3–58.6)	(9.2–22.7)
	*p*-value	0.71	Ref	0.028

Highest coverage per row is highlighted in green, middle coverage values in yellow, and lowest coverage per row in red. CVR, capture-vaccinate-release; HDR, human: dog ratio; SRS, sight re-sight survey.

A two-sample *t*-test indicated no significant difference between CVR and eCVR coverage (*p* = 0.71), confirming comparable performance between the two CVR methods. However, RSP coverage was significantly lower than eCVR coverage (*p* = 0.028), demonstrating that the RSP strategy achieved markedly reduced vaccination reach across sites.

### 3.3 Field survey data reliability

To assess the reliability of post-vaccination SRS data, the proportion of each zone’s total area covered by vaccination routes and SRS transects was calculated and compared (Table 4). Urban sites spanned a substantially smaller geographic area than peri-urban sites (50.2 km^2^ vs. 533.9 km^2^). Consequently, spatial coverage was higher in urban sites, with vaccination routes covering 50.9% of the total area and SRS transects 17.7%, compared with 22.6% and 7.5%, respectively, in peri-urban sites.

**TABLE 4:** Spatial coverage overlap of vaccination routes and SRS transects by site and zone polygon, used to assess post-vaccination data reliability.

Site	Vaccination method	Site area (km^2^)	Vaccination geographic coverage		PVS geographic coverage		PVS-vaccination geospatial overlap		Vaccination coverage reliability rating	Confidence in vaccination coverage estimates
			km^2^	%	km^2^	%	km^2^	%		
Defined	Defined	*R* or Arc	*R* or Arc	Calculation	*R* or Arc	Calculated	*R* or Arc	Calculated from *R* or Arc as the percent of the PVS that overlapped with the Vax Path	Calculated from *R* or Arc as the proportion of spatial coverage over the proportion of spatial overlap	0–0.5 low confidence 0.5–1.0 moderate confidence > 1 high confidence
Chittagong	C-CVR	11.1	5.7	51%	1.8	16%	1.6	88%	0.58	Moderate
Chittagong	C-eCVR	6.5	4.2	65%	1.6	24%	1.3	82%	0.79	Moderate
Chittagong	C-RSP	3.5	2.4	69%	1.1	32%	1.1	93%	0.74	Moderate
Meghna	M-CVR	20.9	6.8	33%	4.8	23%	3.3	69%	0.48	Low, overestimation risk
Meghna	M-eCVR	24.8	8.9	36%	4.1	17%	3.2	78%	0.46	Low, overestimation risk
Meghna	M-RSP	21.8	7.7	35%	2.6	12%	1.8	67%	0.53	Moderate
Narayanganj	N-CVR	9.1	4.3	47%	2.1	23%	1.7	81%	0.58	Moderate
Narayanganj	N-eCVR	13.6	4.8	36%	1.5	11%	1.3	87%	0.41	Low, overestimation risk
Narayanganj	N-RSP	6.3	4.1	65%	0.5	8%	0.5	98%	0.66	Moderate
Sreepur	S-CVR	239.0	44.0	18%	12.4	5%	9.2	74%	0.25	Low, overestimation risk
Sreepur	S-eCVR	190.0	43.6	23%	13.1	7%	9.6	74%	0.31	Low, overestimation risk
Sreepur	S-RSP	37.4	9.4	25%	2.8	8%	1.0	36%	0.69	Moderate
All	Total	584.1	145.9	25%	48.5	8%	35.5	73%	0.34	Low, overestimation risk

SRS, sight re-sight survey; PVS, post-vaccination survey; CVR, standard capture, vaccinate, release; eCVR, enhanced capture, vaccinate, release; RSP, roaming static point.

Despite differences in absolute coverage, the ratio of vaccination route area to SRS transect area was consistent across both urban and per-urban sites (∼3.4: 1), indicating comparable proportional spatial overlap between vaccination routes and SRS transects.

**FIGURE 5:**
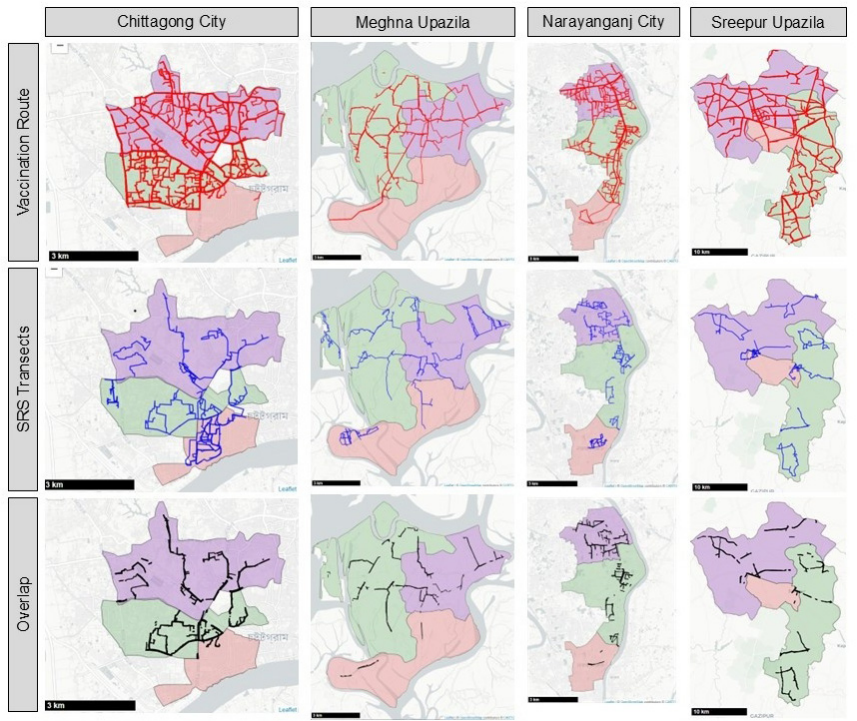
Paths for vaccination routes (red), SRS transects (blue), and spatial overlap (black) across four study sites. SRS, sight re-sight survey.

Spatial overlap (the area shared between vaccination routes and SRS transects) was also higher in urban sites, with 87.4% overlap in Chittagong and 85.5% in Narayanganj, compared to 71.4% in Meghna and 69.9% in Sreepur (Figure 5).

Ideally, post-vaccination surveys should cover a large and representative area without over-representing vaccinated regions, which can bias coverage estimates. Low spatial coverage combined with high overlap may lead to overestimation of coverage, whereas low spatial coverage with low overlap may result in underestimation.

To account for these factors, reliability ratings were calculated for each evaluation zone based on spatial coverage and overlap. Among the 12 zones, seven were rated as moderate reliability (0.5–1.0), while five were rated low (< 0.5), indicating a tendency to overestimate coverage. No zones achieved a high reliability rating (> 1.0). On average, urban sites demonstrated moderate reliability (mean = 0.59), whereas peri-urban sites showed lower reliability (mean = 0.32), reflecting reduced confidence in coverage estimates in these areas. Lower reliability was generally attributed to excessive spatial overlap, limited spatial sampling, or both.

## 4 Discussion

This study evaluated the operational efficiency and effectiveness of MDV campaigns across four sites (two urban and two peri-urban) in Bangladesh using standard CVR, eCVR, and RSP strategies. Among 9,195 dogs vaccinated during 12 campaign days, eCVR was the most efficient and effective, achieving the highest daily vaccination rate (40.2 dogs/vaccinator/day) and estimated coverage (45%; 95% CI: 31.3–58.6). Standard CVR showed slightly lower efficiency (36.6 dogs/vaccinator/day) and coverage (37%) with comparable performance to eCVR (*p* = 0.71), while RSP was markedly less effective (18.2 dogs/vaccinator/day; 16% coverage; *p* = 0.028 vs. eCVR). The elevated eCVR outcomes are consistent with evidence highlighting that smartphone applications can improve team efficiency and facilitate real-time monitoring and evaluation to support MDV program expansion (Gibson et al., 2018; Monroe et al., 2021). However, the lack of a significant difference between eCVR and standard CVR may reflect Bangladesh’s long history of operating vaccination programs, which likely offered vaccinators familiarity with the locations. In previous studies, significant improvement was observed with eCVR, but typically in settings with minimal prior vaccination campaign experience.

Post-vaccination surveys revealed that over 93% of dogs were free-roaming, with approximately 30% unowned, explaining the limited effectiveness of static point strategies (Gibson et al., 2016). Similar observations have been reported previously in Bangladesh and India, where loosely owned, community dogs were less likely to be reached in static point campaigns (Evans et al., 2022; Hossain et al., 2013; Tenzin et al., 2015). Urban sites had higher proportions of unowned dogs (36.7%) than peri-urban sites (17.3%). These findings underscore the need for context-specific vaccination strategies tailored to local dog demographics. Achieving the > 70% herd immunity threshold nationwide in Bangladesh would likely require CVR to reach hard-to-catch dogs, potentially supplemented by oral rabies vaccination (ORV) to improve coverage among elusive or hard-to-handle dogs (Bonwitt et al., 2020; Sánchez-Soriano et al., 2020; Undurraga et al., 2020; Wallace et al., 2020).

Mean vaccination coverage remained below the 70% target, contrasting with past reports of much higher post-vaccination herd immunity (> 80%) (Ghosh et al., 2024). One factor likely contributing to this apparent contradiction is an initial underestimation of HDRs (urban: 73.3, peri-urban: 61.4 vs. planning HDR of 100:1), resulting in more dogs in these communities than what was officially estimated. Additionally, this analysis highlights the risks associated with inappropriately conducted post-vaccination survey methods, which may have affected previous campaign evaluations. Despite extensive training to minimize such bias, low reliability ratings in peri-urban zones illustrate how limited spatial survey coverage combined with high overlap between SRS transects and vaccination routes can inflate coverage estimates. High proportions of unowned dogs and operational challenges, especially in large, low-density peri-urban areas, also likely constrained overall coverage. Future campaigns should expand survey coverage, reduce overlap with vaccination routes, and dynamically adjust field efforts using real-time monitoring, particularly in sparsely populated areas.

HDR estimates varied considerably across sites, consistent with the wide range observed in prior SRS-based studies in Bangladesh [828:1 in urban Dhaka (Tenzin et al., 2015); 120:1 in rural regions (Hossain et al., 2013); national average of 86.7:1 (Ghosh et al., 2024)]. These large discrepancies indicate that reliance on a single national HDR can lead to underestimation and suboptimal campaign planning. Advanced, context-sensitive enumeration methods that incorporate variables such as urbanicity and religion offer a reliable alternative. The Settlement Type and Road Connectivity (STARC) model developed by the IRT provides rapid, data-driven dog population estimates using a dog-density formula (Corbett et al., 2025). In this study, dog density formulas showed strong logarithmic correlations with human population density and both total dog density (*R*^2^ = 0.83) and free-roaming dog density (*R*^2^ = 0.754) (Figure 4), offering a scalable alternative to single-value HDR extrapolation. Coverage estimates differed by enumeration method: 19% (dog density), 32% (HDR), and 47% (SRS). These inconsistencies reflect inherent biases, where SRS may overstate coverage due to route overlap, HDRs may misrepresent heterogeneous populations, and dog density models require expertise for accurate application.

In LMICs, where rabies programs face chronic resource constraints, optimizing campaign efficiency is critical. Post-vaccination evaluations provide essential data for characterizing dog populations and identifying coverage gaps, particularly in first-time campaigns without baseline information. To sustainably control rabies, countries must achieve ≥ 70% coverage of free-roaming dogs; in Bangladesh, this equates to vaccinating over one million dogs annually (Ghosh et al., 2024). Findings from this study offer practical guidance for national-scale planning, including improved spatial sampling protocols and the use of population estimation tools such as the STARC model (Corbett et al., 2025).

Although technical frameworks for rabies elimination are well established, operational challenges persist in complex and dynamic environments. Effective program delivery requires context-specific adaptations that reflect variations in dog ownership, community structure, geography, and logistical capacity. This study demonstrates the value of robust, real-time data in guiding operational decisions and evaluating campaign success. Sustained rabies elimination in Bangladesh will depend on proven MDV delivery models, combined with data-driven strategies that can adapt to local conditions and effectively reach the susceptible dog population.

## Data Availability

The raw data supporting the conclusions of this article will be made available by the authors, without undue reservation.
